# Characterization of cytotoxic activity of compounds derived from anacardic acid, cardanol and cardol in oral squamous cell carcinoma

**DOI:** 10.1186/1753-6561-8-S4-P30

**Published:** 2014-10-01

**Authors:** Lucio Neto, Nayara Matos, Wellington Gonzaga, Luiz Romeiro, Maria Santos, Damaris Santos, Andrea Motoyama

**Affiliations:** 1 University of Brasilia, Brasilia, Brasil

## Background

Cancer is the second leading cause of death worldwide, and oral cancer ranks tenth among all types [1]. Chemotherapy, radiotherapy and surgery are current therapeutic options; however these are not fully efficient. Permanent functional impairment and aesthetic scars are frequent [2]. In this scenario, it is crucial to find therapeutic alternatives, including those derived from the flora, which currently provides about 1/3 of all new medicines. Anacardic acids, cardanols and cardols are the main constituents of the cashew nut shell liquid (herein referred to as "LCC") and together, account for 90% of its composition. The liquid is an industrial by-product, with low economic value prior to processing. The nut, the proper fruit from the plant *Anacardium occidentale*, is edible. Anacardic acids, cardols and cardanols are made of a phenolic ring connected to a long side chain (usually C15H31-n) that can bear several to none insaturations. Additionally, a methyl group can be found in the phenolic ring [3]. Apart from current industrial uses, it has been demonstrated that some of these compounds may exert microbicide and anti-oxidative activities. Anacardic acid has been shown to be cytotoxic to lung, liver and gastric tumor cells through epigenetic mechanisms by inhibiting histone acetyl-transferases (HATs) [4] and in a capase-independent manner [5]. However, given the possible molecular diversity obtained from LCC constituents, not all distinct LCC derivatives have yet been fully analyzed or characterized.

## Aim

The aim of the present study was to screen for compounds with cytotoxic activity in oral cancer cells and characterize the observed effect.

## Methods

Constituents of LCC were extracted, purified and subjected to chemical reactions to generate new compounds. Oral squamous carcinoma cells (OSCC-3) were treated with the parental and derived compounds (total of 8) at 25ng/uL for 24h, 48h and 72h, as well as with staurosporine (300nM) and ethanol (diluent of compounds); as positive and negative controls, respectively. Citotoxicity and cell viability were measured by spectrophotometry and crystal violet assays. To investigate dose-dependency, treatment with compounds with promising results was additionally carried out, at concentrations of 0, 5, 10, and 25ng/mL, and cell viability was measured. In order to identify the type of cell death, DNA fragmentation studies and Western Blot for caspases were performed.

## Results

Of the eight compounds tested, four showed initial cytotoxic activity at 25ng/mL, at all incubation times analyzed. When tested for dose-dependency, two compounds induced, at concentrations between 10 and 25ng/mL, a marked decrease in cell viability, which dropped from approximately 70% to less than 20%. DNA fragmentation assay showed that one compound induced apoptosis, whereas its saturated counterpart did not. These results were further expanded by western blot analyses.

## Conclusion

Compounds derived from LCC have considerable cytotoxic activity towards oral cancer cells. Because of their versatility, it is possible to determine molecular motifs that may mediate these effects. In this light, new therapeutic agents may be developed from the compounds tested.
